# Hyperbaric oxygen therapy can ameliorate the EMT phenomenon in keloid tissue

**DOI:** 10.1097/MD.0000000000011529

**Published:** 2018-07-20

**Authors:** Mingzi Zhang, Shu Liu, Enling Guan, Hao Liu, Xinhang Dong, Yan Hao, Xin Zhang, Pengxiang Zhao, Xuehua Liu, Shuyi Pan, Youbin Wang, Xiaojun Wang, Yifang Liu

**Affiliations:** a Department of Plastic Surgery, Peking Union Medical College Hospital; b Department of Plastic Surgery, China Meitan General Hospital Affiliated to North China University of Science and Technology, Beijing; c Department of Ear-Nose-Throat, Qingdao Huangdao District Hospital of Traditional Chinese Medicine, Qingdao, Shandong; d College of Life Science and Bioengineering, Beijing University of Technology; e Department of Hyperbaric Oxygen, Beijing Chao-Yang Hospital; f Department of Hyperbaric Oxygen, Navy General Hospital; g International education college, Beijing Vocational College of Agriculture, Beijing, China.

**Keywords:** epithelial-to-mesenchymal transition, hyperbaric oxygen therapy, keloid

## Abstract

**Background:**

Hyperbaric oxygen therapy (HBOT) has been widely used in the clinical setting. In this study, HBOT therapy was evaluated for its ability to ameliorate the epithelial-to-mesenchymal transition (EMT) phenomenon in keloid tissue.

**Methods:**

Keloid patients were randomly divided into two groups: keloid patients (K group, 9 patients) and keloid patients receiving HBOT (O group, 9 patients). A third group with normal skin (S group, 9 patients) was established for control. Before HBOT and surgery, a laser Doppler flowmeter was used to measure the keloid blood supply of patients in the O group. Hematoxylin and eosin (H&E) staining was used to observe morphology. E-cadherin, ZO-1, vimentin, fibronectin, vascular endothelial growth factor (VEGF), and hypoxia inducible factor (HIF)-1α were measured by immunofluorescence staining and Western blot analysis. Real-time quantitative polymerase chain reaction (RT-qPCR) was used to evaluate the mRNA expression level of these factors as well.

**Results:**

In the O group, keloid blood perfusion was significantly reduced after patients received HBOT. Compared with the K group, lower expression levels of vimentin, vibronectin, VEGF, and HIF-1α were observed in the O group, whereas the expression of E-cadherin and ZO-1 was significantly higher. The mRNA expression of E-cadherin and ZO-1 was also increased after HBOT.

**Conclusions:**

The expression levels of factors related to the EMT phenomenon were significantly reversed in keloid patients after they received HBOT, indicating that HBOT may be an effective therapy against the EMT phenomenon in keloid patients.

## 1 Introduction

Keloids, or areas of irregular fibrous tissue formed at the site of a scar or injury, are typically regarded as resulting from abnormal wound healing that extends beyond the area of the original skin injury and occurs in predisposed individuals.^[1–4]^ The appearance of a keloid is disfiguring; it can also cause physical discomfort, functional limitation, and significant psychological morbidity,^[5,6]^ thus seriously affecting patients’ quality of life.

The epithelial-to-mesenchymal transition (EMT) phenomenon was first reported by Elizabeth Hay in 1960^[7]^ and was recognized as a feature of embryogenesis.^[8]^ EMT is a process whereby epithelial cells lose their cell polarity and cell-cell adhesion, develop invasive and migratory properties, and ultimately become mesenchymal cells. Epithelial markers such as E-cadherin and zonula occludens-1 (ZO-1) and mesenchymal markers such as vimentin and fibronectin are typically involved in this process. EMT has been shown to occur in wound healing, organ fibrosis, and the development of cancer.^[8]^ Ma et al^[9]^ found that the EMT phenomenon existed in keloid tissue; they demonstrated that a hypoxia/hypoxia-inducible factor-1α (HIF-1α) rich microenvironment favored the transformation of keloid-derived keratinocytes into fibroblast-like cells through the EMT phenomenon, thus supporting the invasive growth of a keloid.

Hyperbaric oxygen therapy (HBOT) is defined as breathing 100% oxygen while under increased atmospheric pressure. In the clinical setting, HBOT has been used in the treatment of challenging wounds and selected neurologic diseases. In the field of plastic surgery, HBOT is also regarded as a successful adjunctive therapy for promoting wound healing, reducing inflammatory reactions, and improving flap survival.^[10,11]^ HBOT also shows a protective effect in many animal models of disease, including stroke^[12]^ and spinal cord injury.^[13]^ In a recent study of murine glioma, HBOT inhibited the proliferation of glioma cells and infiltration inflammatory cells; it also sensitized patients to nimustine therapy, partly by increasing oxygen pressure (PO_2_) in tumor tissues but also by decreasing the expression of HIF-1α, tumor necrosis factor (TNF)-α, interleukin (IL)-1β, vascular endothelial growth factor (VEGF), matrix metalloprotein 9, and nuclear factor kappa-light-chain-enhancer of activated B cells.^[14]^ The present study was designed to determine whether HBOT had an effect against the EMT phenomenon and to do so by measuring and assessing the expression levels of EMT-related markers.

## 2 Methods

### 2.1 Hyperbaric oxygen treatment procedure

Patients underwent hyperbaric oxygen treatment for 7 days before surgery (once a day, 7 times in total) in a medical hyperbaric chamber with 3 locks and 7 doors pressurized with air. Pressure was increased to 0.2 MPa (2 ATA) at a constant speed within 30 minutes. Then, patients inhaled 100% oxygen through face masks. After 60 minutes of inhalation, pressure was decreased to normal within another 30 minutes. Surgery was performed 24 hours later after the last HBOT.

### 2.2 Patients and grouping

The clinical study protocol was reviewed and approved by the Bioethical Committee of Peking Union Medical College Hospital in Peking, China. All methods were performed in accordance with the relevant guidelines and regulations. Written informed consent was given by all patients. In all, 27 patients (18 keloid patients and 9 nonkeloid patients) were randomly selected from the department of plastic surgery at Peking Union Medical College Hospital from January to December 2016. Keloid patients were randomly divided into 2 groups: keloid patients (K group) and keloid patients with HBOT (O group). A third group with normal skin (S group) was established for control. There were no significant differences in the keloid baseline (melanin, height, vascularity, pliability, and total score) between the K and O groups, which were evaluated according to the Vancouver scar scale (VSS). There were 5 male patients and 4 female patients in each group; their ages ranged from 18 to 50 years (median age: K: 31; O: 32; S: 32). All samples were taken in the chest region. Keloid samples were taken from the centers of the keloid tissues. No significant differences in age, sex, or site were observed between the 2 keloid groups (*P* > .05). All keloids, caused by trauma, were diagnosed and confirmed by an experienced plastic surgeon and through pathologic examination. No patients had any systemic disorders, were taking drugs, or were receiving other treatments that might affect the study results.

For further analysis, specimens from each patient were cut into 3 parts: for hematoxylin and eosin (H&E) staining and immunofluorescence analysis, one part was placed in a 10% formalin solution for paraffin embedding; for Western blot studies, one part was stored in liquid nitrogen immediately after excision; and for real-time quantitative polymerase chain reaction (RT-qPCR), the third part was immersed quickly in RNA extraction solution for storage Table 1.

**Table 1:**
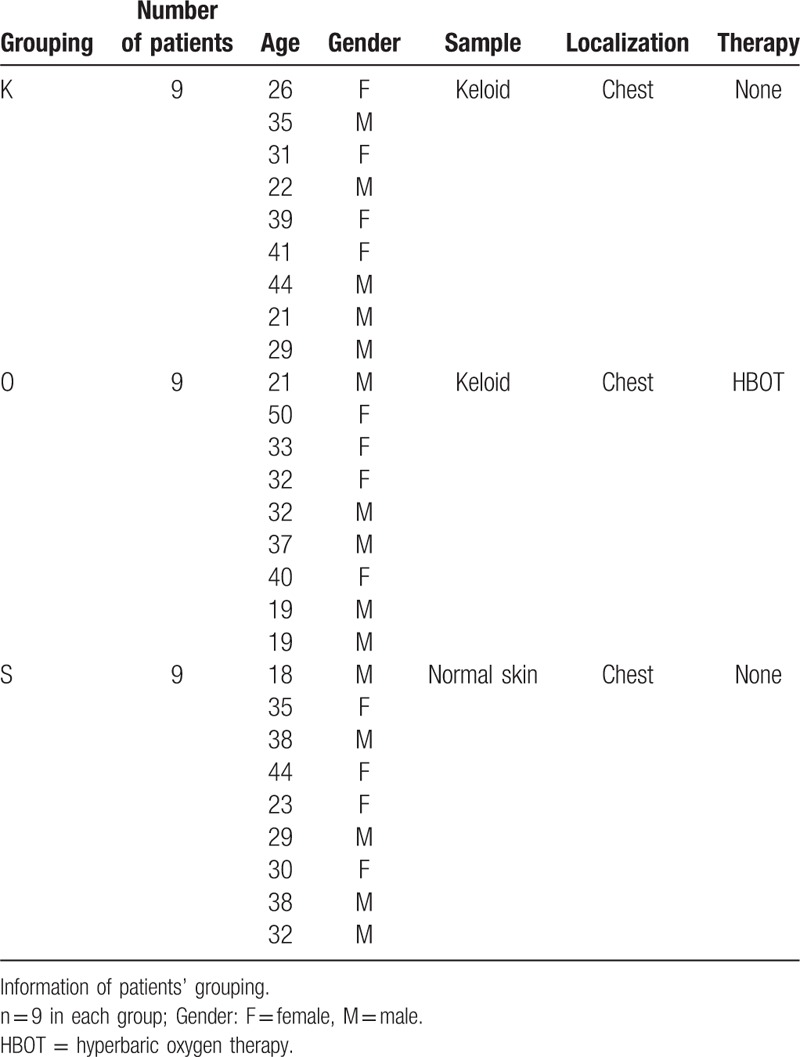
The patients information in each group.

### 2.3 Blood perfusion evaluation of keloid tissue

Patients in the O group received evaluation of keloid tissue blood perfusion twice (24 hours before the first HBOT and 24 hours after the last HBOT, just before surgery) via laser Doppler flowmeter (LDF; Perimed AB, Stockholm, Sweden) and laser speckle contrast analysis (LSCA; Perimed AB, Stockholm, Sweden). The detection area was marked along the edge of the keloid tissue. Patients were required to rest for at least 30 minutes before blood perfusion measurement, keeping calm and in a supine position during the whole process. The distance between the laser scanning probe and the patient was 15 cm. The image acquisition rate was 3 Hz, and the whole process lasted for 3 minutes. The ambient temperature was maintained between 22°C and 25°C. Vascular flow was measured using perfusion units (PUs, mL/100 g/min). The measurement was repeated 3 times for each patient and the average data of the 3 measurements were used for analysis.

### 2.4 H&E staining and immunofluorescence analysis

After being fixed in a 10% formalin solution for 48 hours, specimens were embedded in paraffin, sectioned, and put on slides. Histologic characteristics were observed by H&E staining. As for immunofluorescence analysis, slides were incubated with anti-E-cadherin (1:30; Abcam, Cambridge, UK), anti-ZO-1 (1:100; Abcam), anti-vimentin (1:200; Abcam), anti-fibronectin (1:200; Abcam), anti-VEGF (1:200; Abcam), or anti-HIF-1α (1:200; Abcam) antibodies in a humidified chamber at 4°C overnight (12–16 hours). Primary antibody was marked by goat anti-rabbit IgG H&L (DyLight488) (1:50; Abcam) for 1 hour at room temperature. The slides were subsequently rinsed 3 times with phosphate buffered solution for 5 minutes each. Specimens were stained with 10 mg/mL Hoechst 33258 (Sigma-Aldrich, San Francisco, State of California) for 10 minutes at room temperature to counterstain DNA. A Zeiss Axiophot fluorescence microscope (Axio-Cam MRc; Zeiss, Pberkochen, Germany) with a digital video camera and Axiovision Zeiss software were used to observe the expression of markers listed above. Green areas represent tissue with positive expression of targeted protein; the shade of each green area represents the expression level of the target protein. Blue areas represent areas of DNA. Each sample for a single factor was stained on 3 slides for analysis. In total, 486 (3 slides × 27 patients × 6 factors) slides were subjected to immunofluorescence analysis.

### 2.5 Western blot analysis

A cell lysis kit (Bio-Rad laboratories, Hercules, CA) was used to extract protein from 50-mg samples according to the manufacturer's instructions. Samples were ground on ice for 10 minutes in buffer (246 μL of lysis buffer, 1.25 μL of phosphatase inhibitor, 0.25 μL of protease inhibitor, and 2.5 μL of phenylmethanesulfonyl fluoride (PMSF) and then centrifuged (at 14,000 rpm) at 4°C for 15 minutes. Equal amounts of supernatant protein (60 μg) were separated by 10% SDS-PAGE and transferred to nitrocellulose membranes for immunoblotting. The Western blot membrane was blocked with blocking buffer (Li-cor, Lincoln, NB) for 2 hours and then incubated with anti-E-cadherin (1:500; Abcam), anti-ZO-1 (1:500; Abcam), anti-vimentin (1:500, Abcam), anti-fibronectin (1:500; Abcam), anti-VEGF (1:500; Abcam), or anti-HIF-1α (1:500; Abcam) antibodies overnight for 12 to 16 hours at 4°C. The membranes were incubated with secondary antibodies (Li-cor, Lincoln, NB) at a 1:10,000 dilution for 1 hour at room temperature in the dark. A double-color infrared laser imaging system (Odyssey, Li-cor) was used for detection.

### 2.6 RNA isolation and RT-qPCR

Total RNA was extracted from 30-mg samples with the RNeasy Fibrous Tissue Mini Kit (Qiagen, Düsseldorf, Germany) according to the manufacturer's instructions. A UV spectrophotometer (Thermo, Waltham, MA) was used to measure the concentration of the extracted RNA, which was visualized via 1% agarose gel electrophoresis. Reverse transcription of 1 μg of total RNA for cDNA synthesis was performed using the ProtoScript M-MuLV First Strand cDNA Synthesis Kit (New England Biolabs, Ipswich, MA), using an anchored oligo-d(T) primer (d[T]23VN). A real-time qPCR kit [Maxima SYBR Green /ROX qPCR Master Mix (2X); ThermoFisher Scientific, Waltham, MA] was used to perform amplification and quantification. The primers for human E-cadherin, ZO-1, vimentin, fibronectin, VEGF, HIF-1α, and β-actin are summarized in Table 2. The expression of target genes was normalized using the β-actin gene as a control. Real-time qPCR cycle parameters included initial denaturation at 95°C for 10 minutes followed by 40 cycles of denaturation at 95°C for 15 seconds, annealing at 55°C for 30 seconds, and extension at 72°C for 30 seconds.

**Table 2:**
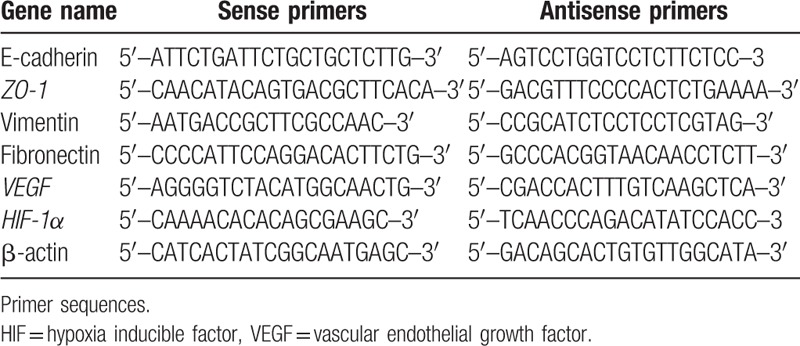
The primer sequences used in this study.

### 2.7 Statistical analysis

Data are presented as the mean ± standard deviation (mean ± SD). SPSS Statistics 24.0 software (SPSS, Inc., Chicago, IL) was used for statistical analysis. The paired samples *t* test was used for blood perfusion analysis in the O group. One-way analysis of variance (ANOVA) followed by the LSD *t* test was used for other statistical analyses. A *P* value of <.05 was considered statistically significant.

## 3 Results

### 3.1 Clinical appearances and pathology findings of keloid compared with normal skin

**Figure 1:**
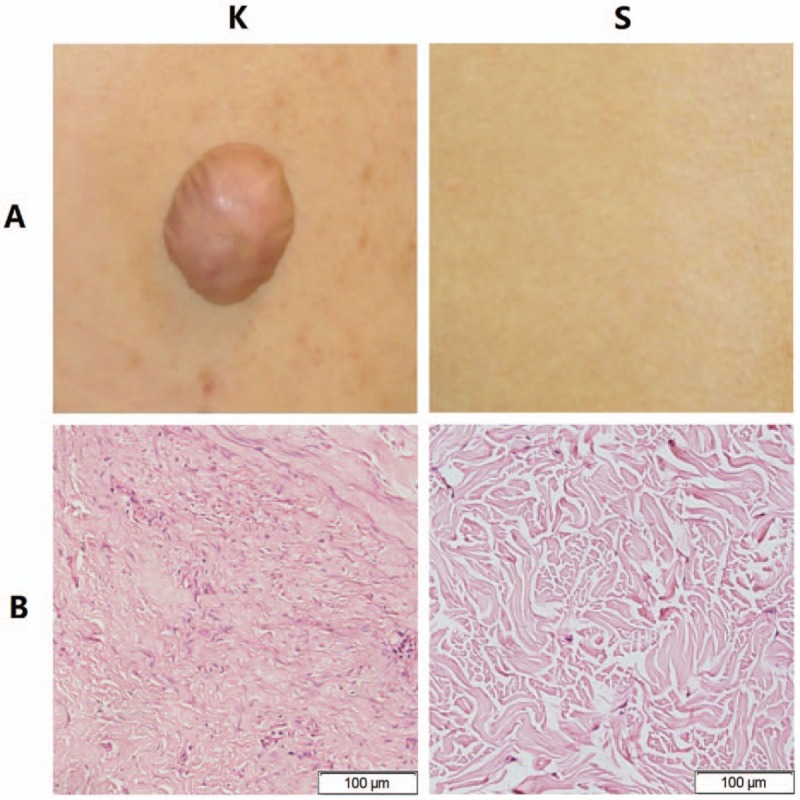
The appearance and morphology of keloid tissue and normal skin. (A–K) Keloid tissue usually appears as firm broad nodules, often erythematous and with a shiny surface. (A–S) Normal skin tissue. (B–K) The morphology of keloid tissue by hematoxylin and eosin (H&E) staining (images: 400 × ). Fibroblasts with more cytoplasm and clear nucleoli are observed, and collagen fibrils appear disordered. (B–S) The morphology of normal skin tissue by H&E staining (images: 400 × ). Collagen fibrils appear relatively loose and orderly.

Keloids usually appear as firm broad nodules, often erythematous, with a shiny surface and occasional telangiectasis. The scar tissue usually extends in a claw-like pattern beyond the area of the initial skin injury^[1]^ (Fig. 1A–K). H&E-stained tissue was used to confirm the pathologic examination. Multiple fibroblasts and intensive inflammatory infiltration were observed. Thick and extremely compact collagen fibrils appeared disordered^[15]^ (Fig. 1B–K). Fig. 1 A to S and B to S show the appearance and histologic morphology of normal skin tissue, where collagen fibrils are arranged in an orderly pattern.

### 3.2 The blood perfusion of keloid tissue decreased after HBOT

**Figure 2:**
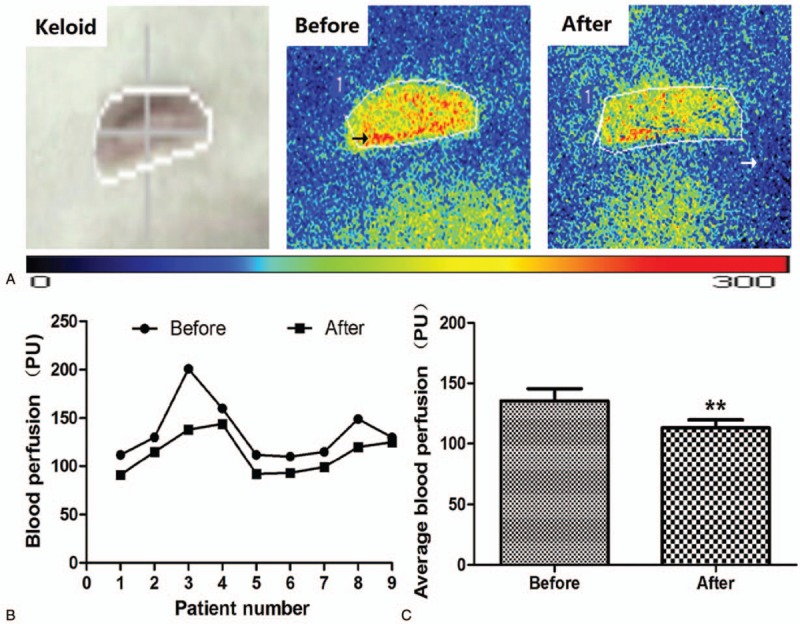
Blood perfusion of keloid tissue before and after HBOT. The black arrow points to red and yellow areas representing tissue richly perfused with blood; the white arrow points to adjacent green or blue areas representing tissue with normal blood perfusion. (A) Representative photographs showing the microcirculation of keloid tissue in patients who received HBOT. (B, C) The average blood perfusion of keloid tissue. Blood perfusion decreased significantly after HBOT. Values shown as mean ± SD (n = 9 in each group; ^∗^*P* < .01).

Keloid patients in the O group received HBOT for 7 days before surgery. The average blood perfusion of keloid tissue before HBOT was 135.4444 ± 30.2246 PU (mL/100 g/min), which was decreased following HBOT (113.0000 ± 20.3224 PU). The difference in average blood perfusion before and following HBOT treatment was statistically significant (*P* < .01) (Fig. 2).

### 3.3 Decreased expression of HIF-1α and VEGF in keloid tissue after HBOT

**Figure 3:**
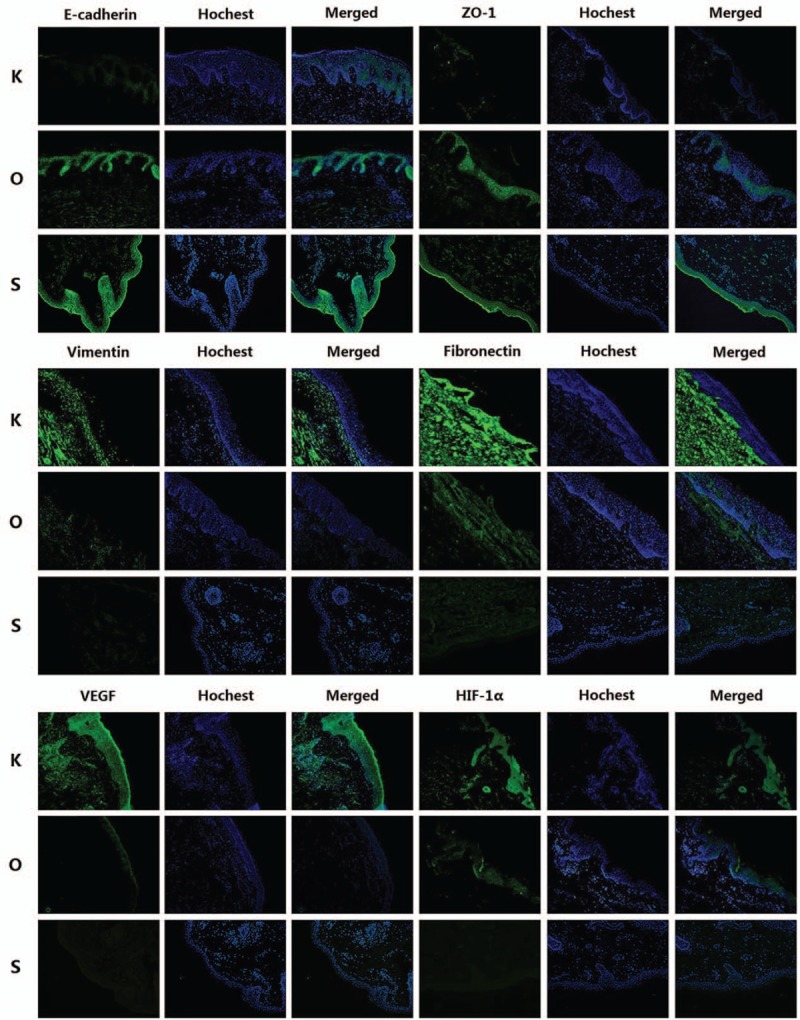
Images (200×) of immunofluorescence staining for all factors. Green areas represent tissue with high expression of target protein, whereas blue areas represent areas of DNA. The expression of vimentin, fibronectin, VEGF, and HIF-1α was decreased in the O group, whereas the expression of E-cadherin and ZO-1 was increased after HBOT (n = 9 in each group). HIF = hypoxia inducible factor, VEGF = vascular endothelial growth factor.

**Figure 4:**
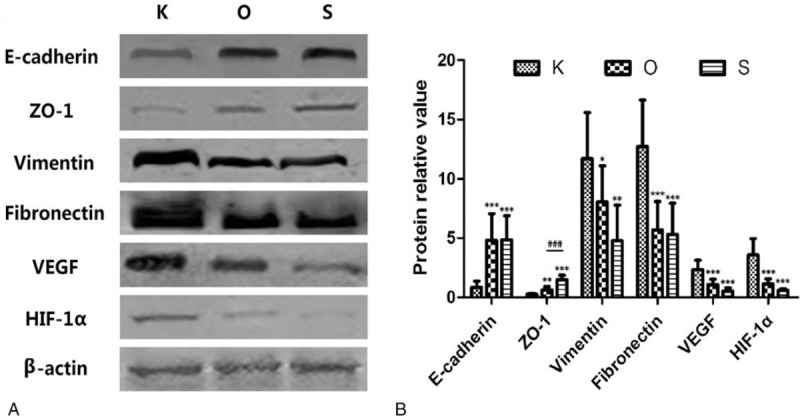
Relative protein amounts for all target proteins. (A) Representative images of Western blots for E-cadherin, ZO-1, vimentin, fibronectin, VEGF, and HIF-1α. (B) The results of densitometry analysis of target proteins, which were consistent with the immunofluorescence analyses. Values shown as mean ± SD (n = 9 in each group, ^∗^*P* < .05, ^†^*P* < .01, ^‡^*P* < .001; in the K group, ^§^*P* < .001). HIF = hypoxia inducible factor, VEGF = vascular endothelial growth factor.

**Figure 5:**
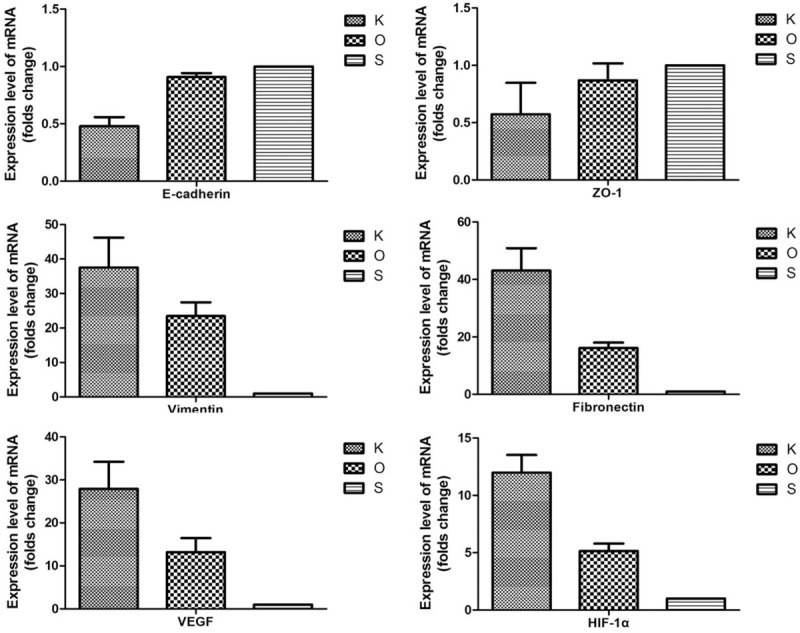
Analysis of the mRNA expression levels of target factors. Compared with the K group, E-cadherin and ZO-1 are highly increased in the O group; however, vimentin, fibronectin, VEGF, and HIF-1α are decreased. Values shown as mean ± SD (n = 9 in each group). HIF = hypoxia inducible factor, VEGF = vascular endothelial growth factor.

**Figure 6:**
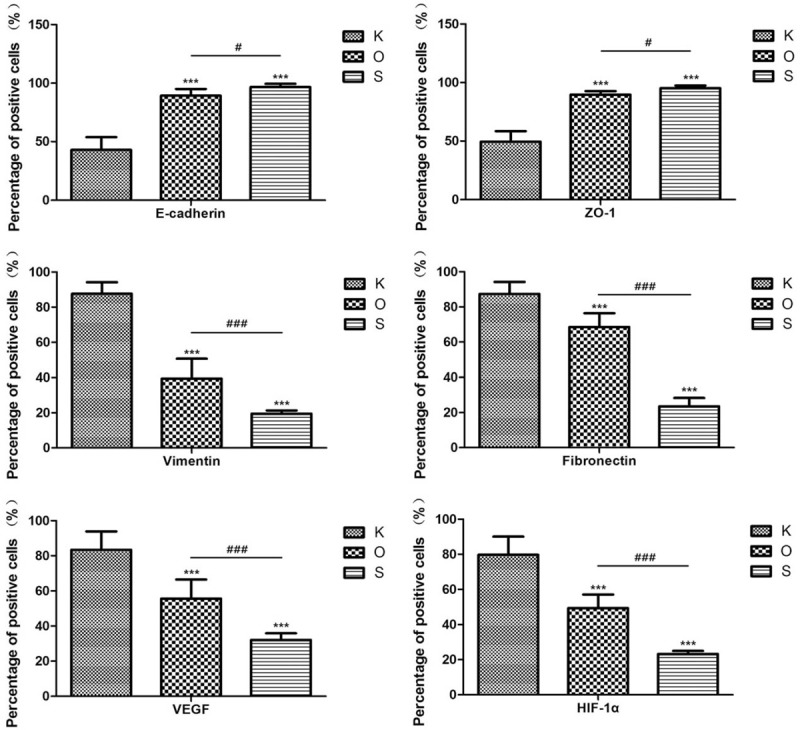
The percentage of positive cells in all groups. Vimentin, fibronectin, VEGF, and HIF-1α showed higher expression and positive cells in the K group. Values shown as mean ± SD (n = 9 in each group; ^∗^*P* < .001; in the K group, ^†^*P* < .05; ^‡^*P* < .001). HIF = hypoxia inducible factor, VEGF = vascular endothelial growth factor.

Qualitative analysis of E-cadherin, ZO-1, vimentin, fibronectin, VEGF, and HIF-1α expression was determined by immunofluorescence and Western blot studies. The protein (Figs. 3 and 4, Table 3) and mRNA expression (Fig. 5 and Table 4) of HIF-1α and VEGF was more obviously greater in the K group than in the O and S groups. Table 5 and Fig. 6 show the percentage of positively stained cells as analyzed via immunofluorescence. There is a significant difference for VEGF and HIF-1α between the K and O groups (*P* < .001).

**Table 3:**
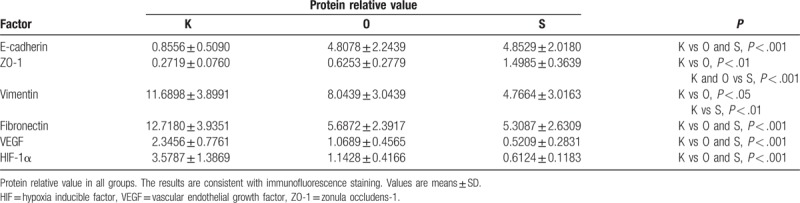
Protein relative value of all markers in each group.

**Table 4:**
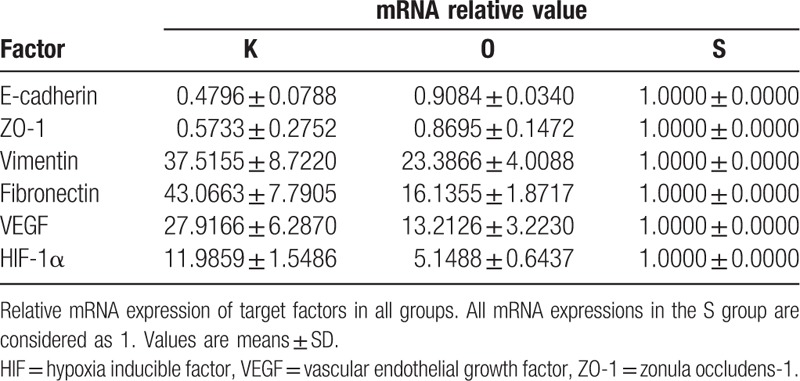
mRNA relative value of all markers in each group.

**Table 5:**
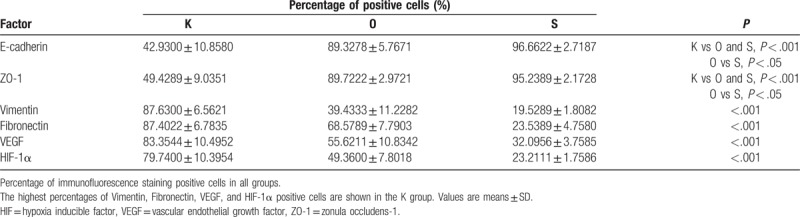
Percentage of positive cells in immunofluorescence staining analysis of each group.

### 3.4 Changed expression of epithelial and mesenchymal markers in keloid tissue after HBOT

The expression of mesenchymal markers (vimentin and fibronectin) was higher in the K group than in the O and S groups (Fig. 3). The results of Table 5 and Fig. 6 also demonstrated a significant difference for all epithelial and mesenchymal markers between the K and O groups (*P* < .001). Consistent with the immunofluorescence staining result, Western blot analysis (Fig. 4 and Table 3) showed higher expression of E-cadherin and ZO-1 in the O group, whereas vimentin and fibronectin revealed the opposite results, with higher expression in the K group. The mRNA expression showed the same results (Fig. 5 and Table 4). Mesenchymal markers displayed the highest expression level among all the tested factors. Although the overall expression of E-cadherin and ZO-1 was relatively low, there was a significant difference between the K and O groups.

## 4 Discussion

Keloids are formed following trauma to the skin and usually occur in predisposed individuals. They are also considered to be a result of irregular wound healing, with excessive formation of scar tissue extending beyond the area of the initial wound, which does not recover spontaneously.^[2–4]^ Keloids commonly occur in the chest, shoulder, or earlobe regions, and the morphologic appearance of keloids may vary in different anatomic areas.^[16,17]^ The continuous formation of keloids can cause significant discomfort, with pain or pruritus. In the study of Lee et al,^[18]^ 46% of patients noted keloid-associated pain and 86% noted pruritus. Darker-skinned individuals develop keloids after trauma approximately 15 times more often than lighter-skinned individuals. However, the mechanism of keloid pathogenesis remains unclear. In recent studies, specific factors (Chemokine-like factor-1,^[15]^ endothelin 1,^[19]^ and growth/differentiation factor 9^[20]^), several genes (*p53*^[21]^ and *Stat-3*^[22]^), and various types of human leukocyte antigens (HLAs)^[23–26]^ (HLA-DR5, HLA-DQ23, HLA-DQA1, and HLA-DQB1) were found to be involved. Keloids can easily recur after monotherapy, including surgical excision. Combined approaches are therefore more effective therapeutic strategies.^[1,2,4]^

EMT is a phenomenon whereby epithelial cells lose cell polarity and cell–cell adhesion and develop invasive and migratory properties. Typical cell markers (epithelial and mesenchymal markers) play a significant role in identifying this process, which is marked by the higher expression of mesenchymal markers (vimentin and fibronectin) and lower expression of epithelial markers (E-cadherin and ZO-1).^[27,28]^ EMT is required for several early processes in embryonic development, including formation of the mesoderm and neural tube.^[29,30]^ Ma et al^[9]^ demonstrated that the EMT phenomenon exists in keloid tissue and that, under hypoxic conditions, HIF-1α upregulates the expression of vimentin and fibronectin and downregulates the expression of E-cadherin and ZO-1 in keloid keratinocytes. That is, low levels of oxygen promote the EMT phenomenon and enhance the invasive ability of keloid keratinocytes.^[9]^ According to that report, HIF-1α may be a key target of this process. In addition, previous studies have reported a hypoxic environment within keloid tissue.^[31,32]^ In a hypoxic environment, HIF-1α is highly expressed and is an important regulator that helps cells adapt to the hypoxic microenvironment.^[33,34]^ Further studies have reported that the stable accumulation of HIF-1α promotes fibrogenesis in many types of tumors through EMT.^[35–38]^ In a hypoxic/HIF-1α rich environment, VEGF is also highly expressed, as it improves the formation of new vessels in tissue.^[31]^

Many therapeutic gases have been used in clinical work, among which HBOT is the best known. That is, 100% oxygen at greater than atmospheric pressure in a treatment chamber is widely used to help heal challenging wounds and intervene in selected neurologic diseases. In the plastic surgery field, postoperative HBOT is commonly used to improve wound healing after flap transfer, with satisfactory results. There has recently been research regarding the use of HBOT as a preconditioning therapy. Cheng et al,^[12]^ for example, reported that HBOT reduced the expression of cyclooxygenase-2 and provided brain protection following ischemia. Other typical uses for HBOT include the treatment of extensive ulcers,^[39]^ including diabetic foot ulcers,^[40]^ flap^[41]^ and other organ ischemia, and reperfusion injuries.^[42–44]^ Hyperbaric oxygen is used because it helps to increase the availability oxygen. Oxygen availability at the tissue level is essential for physiologic function. During HBOT, cellular oxygen concentrates in the plasma, and tissue oxygenation is increased.^[45]^ HBOT has been reported as an effective way of increasing pO_2_ values in tissue.

Because hypoxia/HIF-1α may be a key environmental factor in the EMT process affecting keloid, HBOT might potentially reverse the hypoxic condition and thus also make it possible to ameliorate the EMT phenomenon. In this study, 3 groups were established and samples from 27 keloid patients were analyzed to evaluate the effect of HBOT on the EMT phenomenon. H&E staining was performed to analyze keloid morphology. Fibroblasts with more cytoplasm and clear nucleoli, disordered collagen fibrils, and infiltrated cells were observed in the keloid tissue. Patients in the O group had average blood perfusion measurements before undergoing HBOT and before surgery. The results showed that the average blood perfusion of keloid tissue was significantly decreased after 7 days of HBOT. If oxygen is administered to support wound healing, it can accelerate and sustain vessel growth.^[46]^ If this is the case, why did average blood perfusion decrease after HBOT? According to Steinbrech et al,^[31]^ the hypoxic environment induced a high expression of VEGF, which improved angiogenesis in coping with the increased need for oxygen to support the metabolism of keloid tissue. After receiving regular HBOT, pO_2_ was effectively increased in keloid tissue; more oxygen was dissolved in each unit of blood, so that less blood was required overall. Analyses of the expression levels of E-cadherin, ZO-1, vimentin, fibronectin, VEGF, and HIF-1α via immunofluorescence and Western blot are quite similar. In the K group, higher expression of vimentin, fibronectin, VEGF, and HIF-1α as well as lower expression of E-cadherin and ZO-1 were observed, consistent with the reports of Ma et al^[9]^ and Steinbrech et al.^[31]^ The pattern of expression of target markers was reversed in the O group. The results of mRNA expression analysis revealed that HBOT remarkably increased the level of E-cadherin and ZO-1 mRNA; in contrast, mRNA expression of other markers was significantly inhibited.

On the basis of these results, HBOT effectively ameliorated the EMT condition of keloid tissue. This effect may be caused by an increase of oxygen within keloid tissue due to HBOT. That is, an oxygen-rich environment meets the need of high keloid metabolism and reverses the expression of HIF-1α and VEGF, causing decreased blood perfusion. Because of the low expression of HIF-1α, EMT-related markers also changed; the expression of epithelial markers was low and that of mesenchymal markers was high.

HBOT significantly decreased the expression levels of vimentin, fibronectin, and HIF-1α, suggesting that HBOT has protective effects against the EMT phenomenon during keloid development. In addition, the average blood perfusion and VEGF levels of keloid tissue were remarkably decreased after HBOT, indicating that this may be a new way of ameliorating the appearance and color of keloid tissue. However, our study focused only on HBOT's effect on keloid tissue; thus, it did not clarify the mechanism of this process or the role of HIF-1α. Furthermore, the effect of HBOT on the EMT phenomenon in other body parts and in patients of different ages requires further study.

## 5 Conclusion

In this study, keloid tissue after HBOT demonstrated lower expression levels of vimentin, fibronectin, VEGF, and HIF-1α. These results indicate that HBOT can ameliorate the hypoxic microenvironment of keloid tissue and that it has protective effects against the EMT phenomenon.

## Author contributions

Y.W. conceived the study and modified the paper. M.Z and S.L. wrote the main manuscript text. M.Z and S.L. are cofirst authors. S.L, M.Z, E.G, H.L., and X.D. performed the experiments and collected data. X.Z. and P.Z. did the statistical analysis. X.L., S.P., and Y.L. performed the HBOT. All authors read and approved the final manuscript.

**Conceptualization:** Yan Hao.

**Data curation:** Enling Guan, Hao Liu, Yan Hao, Yifang Liu.

**Formal analysis:** Xinhang Dong.

**Investigation:** Xin Zhang.

**Project administration:** Shu Liu, Pengxiang Zhao.

**Software:** Xuehua Liu.

**Supervision:** Shuyi Pan.

**Writing – original draft:** Mingzi Zhang.

**Writing – review & editing:** Mingzi Zhang, Youbin Wang, Xiaojun Wang.
